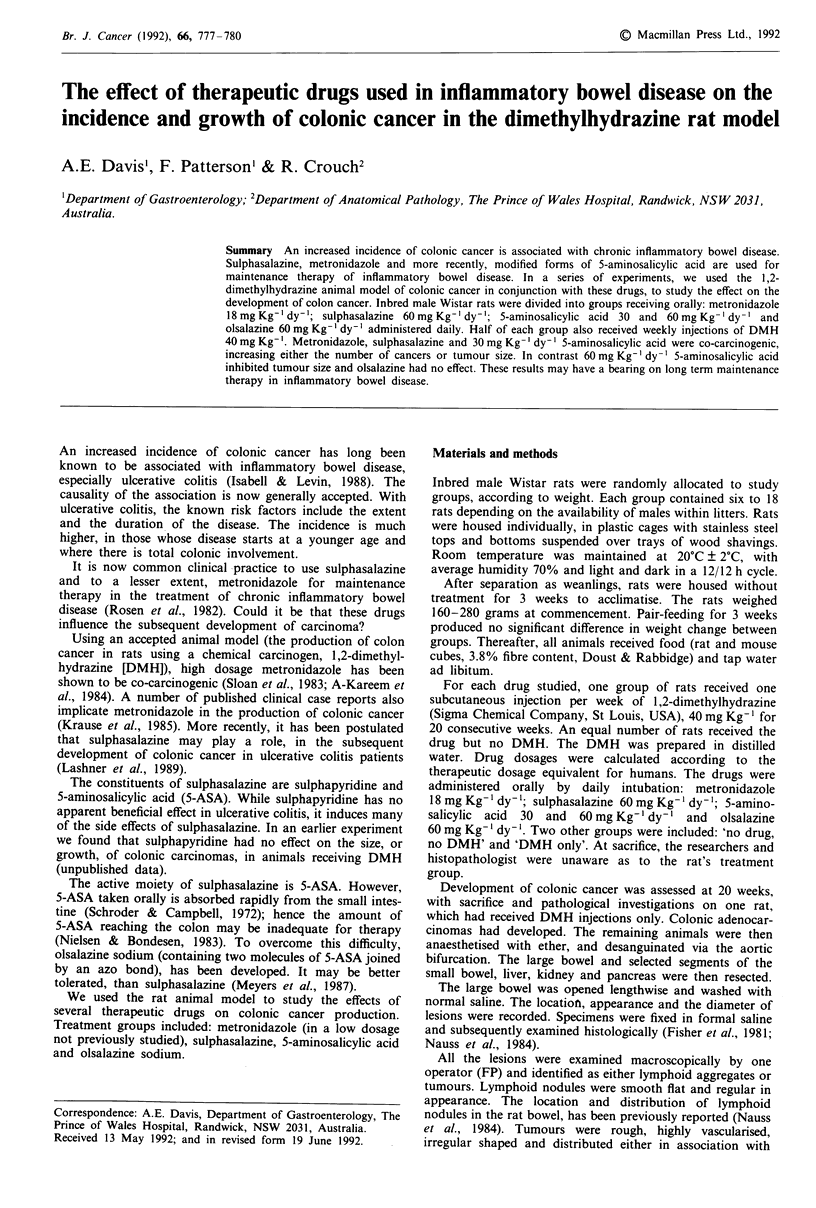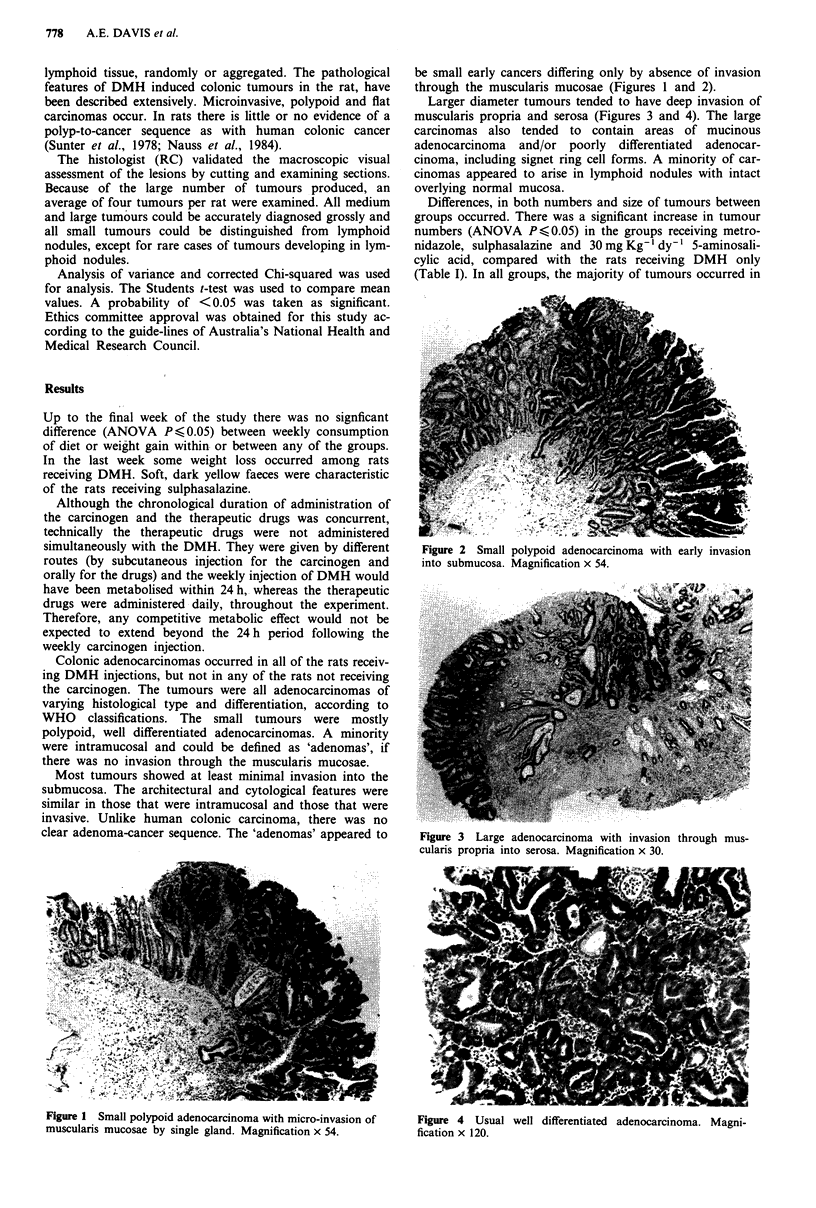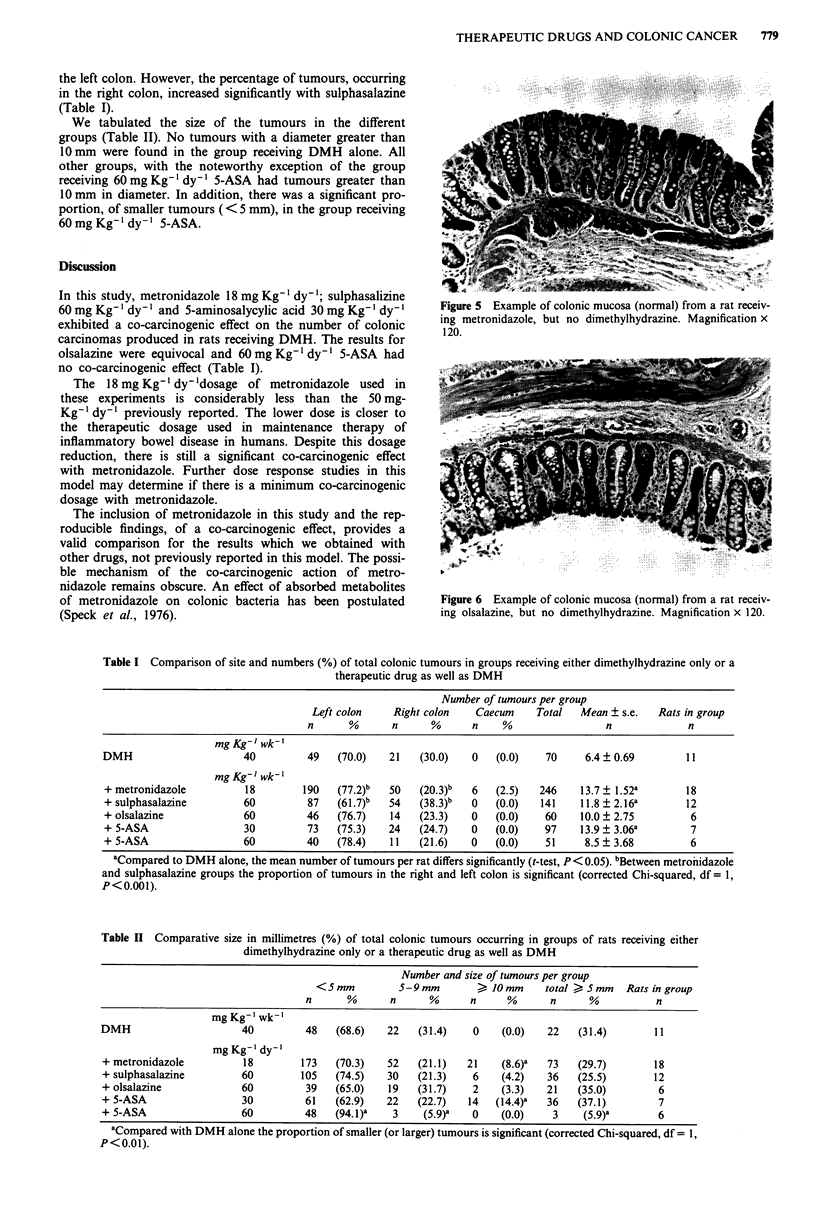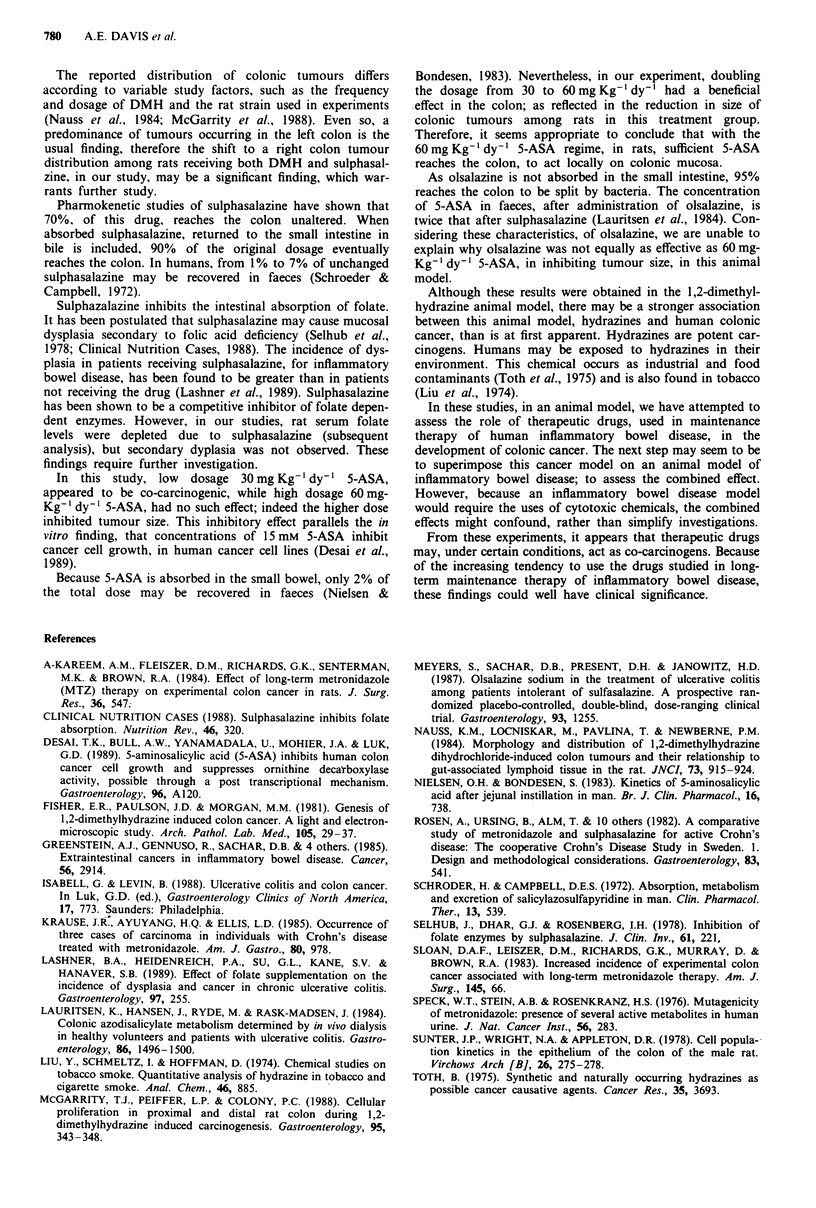# The effect of therapeutic drugs used in inflammatory bowel disease on the incidence and growth of colonic cancer in the dimethylhydrazine rat model.

**DOI:** 10.1038/bjc.1992.359

**Published:** 1992-11

**Authors:** A. E. Davis, F. Patterson, R. Crouch

**Affiliations:** Department of Gastroenterology, Prince of Wales Hospital, Randwick, NSW, Australia.

## Abstract

**Images:**


Br. J. Cancer (1992), 66, 777–780 © Macmillan Press Ltd., 1992

### The effect of therapeutic drugs used in inflammatory bowel disease on the incidence and growth of colonic cancer in the dimethylhydrazine rat model

A.E. Davis[1], F. Patterson[1] & R. Crouch[2]

[1]*Department of Gastroenterology;* [2]*Department of Anatomical Pathology, The Prince of Wales Hospital, Randwick, NSW 2031, Australia.*

**Summary** An increased incidence of colonic cancer is associated with chronic inflammatory bowel disease. Sulphasalazine, metronidazole and more recently, modified forms of 5-aminosalicylic acid are used for maintenance therapy of inflammatory bowel disease. In a series of experiments, we used the 1,2-dimethylhydrazine animal model of colonic cancer in conjunction with these drugs, to study the effect on the development of colon cancer. Inbred male Wistar rats were divided into groups receiving orally: metronidazole 18 mg $Kg^{-1}$ $dy^{-1}$; sulphasalazine 60 mg $Kg^{-1}$ $dy^{-1}$; 5-aminosalicylic acid 30 and 60 mg $Kg^{-1}$ $dy^{-1}$ and olsalazine 60 mg $Kg^{-1}$ $dy^{-1}$ administered daily. Half of each group also received weekly injections of DMH 40 mg $Kg^{-1}$. Metronidazole, sulphasalazine and 30 mg $Kg^{-1}$ $dy^{-1}$ 5-aminosalicylic acid were co-carcinogenic, increasing either the number of cancers or tumour size. In contrast 60 mg $Kg^{-1}$ $dy^{-1}$ 5-aminosalicylic acid inhibited tumour size and olsalazine had no effect. These results may have a bearing on long term maintenance therapy in inflammatory bowel disease.

An increased incidence of colonic cancer has long been known to be associated with inflammatory bowel disease, especially ulcerative colitis (Isabell & Levin, 1988). The causality of the association is now generally accepted. With ulcerative colitis, the known risk factors include the extent and the duration of the disease. The incidence is much higher, in those whose disease starts at a younger age and where there is total colonic involvement.

It is now common clinical practice to use sulphasalazine and to a lesser extent, metronidazole for maintenance therapy in the treatment of chronic inflammatory bowel disease (Rosen *et al.*, 1982). Could it be that these drugs influence the subsequent development of carcinoma?

Using an accepted animal model (the production of colon cancer in rats using a chemical carcinogen, 1,2-dimethylhydrazine [DMH]), high dosage metronidazole has been shown to be co-carcinogenic (Sloan *et al.*, 1983; A-Kareem *et al.*, 1984). A number of published clinical case reports also implicate metronidazole in the production of colonic cancer (Krause *et al.*, 1985). More recently, it has been postulated that sulphasalazine may play a role, in the subsequent development of colonic cancer in ulcerative colitis patients (Lashner *et al.*, 1989).

The constituents of sulphasalazine are sulphapyridine and 5-aminosalicylic acid (5-ASA). While sulphapyridine has no apparent beneficial effect in ulcerative colitis, it induces many of the side effects of sulphasalazine. In an earlier experiment we found that sulphapyridine had no effect on the size, or growth, of colonic carcinomas, in animals receiving DMH (unpublished data).

The active moiety of sulphasalazine is 5-ASA. However, 5-ASA taken orally is absorbed rapidly from the small intestine (Schroder & Campbell, 1972); hence the amount of 5-ASA reaching the colon may be inadequate for therapy (Nielsen & Bondesen, 1983). To overcome this difficulty, olsalazine sodium (containing two molecules of 5-ASA joined by an azo bond), has been developed. It may be better tolerated, than sulphasalazine (Meyers *et al.*, 1987).

We used the rat animal model to study the effects of several therapeutic drugs on colonic cancer production. Treatment groups included: metronidazole (in a low dosage not previously studied), sulphasalazine, 5-aminosalicylic acid and olsalazine sodium.

Correspondence: A.E. Davis, Department of Gastroenterology, The Prince of Wales Hospital, Randwick, NSW 2031, Australia.
Received 13 May 1992; and in revised form 19 June 1992.

### Materials and methods

Inbred male Wistar rats were randomly allocated to study groups, according to weight. Each group contained six to 18 rats depending on the availability of males within litters. Rats were housed individually, in plastic cages with stainless steel tops and bottoms suspended over trays of wood shavings. Room temperature was maintained at 20°C ± 2°C, with average humidity 70% and light and dark in a 12/12 h cycle.

After separation as weanlings, rats were housed without treatment for 3 weeks to acclimatise. The rats weighed 160–280 grams at commencement. Pair-feeding for 3 weeks produced no significant difference in weight change between groups. Thereafter, all animals received food (rat and mouse cubes, 3.8% fibre content, Doust & Rabbidge) and tap water ad libitum.

For each drug studied, one group of rats received one subcutaneous injection per week of 1,2-dimethylhydrazine (Sigma Chemical Company, St Louis, USA), 40 mg $Kg^{-1}$ for 20 consecutive weeks. An equal number of rats received the drug but no DMH. The DMH was prepared in distilled water. Drug dosages were calculated according to the therapeutic dosage equivalent for humans. The drugs were administered orally by daily intubation: metronidazole 18 mg $Kg^{-1}$ $dy^{-1}$; sulphasalazine 60 mg $Kg^{-1}$ $dy^{-1}$; 5-aminosalicylic acid 30 and 60 mg $Kg^{-1}$ $dy^{-1}$ and olsalazine 60 mg $Kg^{-1}$ $dy^{-1}$. Two other groups were included: 'no drug, no DMH' and 'DMH only'. At sacrifice, the researchers and histopathologist were unaware as to the rat's treatment group.

Development of colonic cancer was assessed at 20 weeks, with sacrifice and pathological investigations on one rat, which had received DMH injections only. Colonic adenocarcinomas had developed. The remaining animals were then anaesthetised with ether, and desanguinated via the aortic bifurcation. The large bowel and selected segments of the small bowel, liver, kidney and pancreas were then resected.

The large bowel was opened lengthwise and washed with normal saline. The location, appearance and the diameter of lesions were recorded. Specimens were fixed in formal saline and subsequently examined histologically (Fisher *et al.*, 1981; Nauss *et al.*, 1984).

All the lesions were examined macroscopically by one operator (FP) and identified as either lymphoid aggregates or tumours. Lymphoid nodules were smooth flat and regular in appearance. The location and distribution of lymphoid nodules in the rat bowel, has been previously reported (Nauss *et al.*, 1984). Tumours were rough, highly vascularised, irregular shaped and distributed either in association with lymphoid tissue, randomly or aggregated. The pathological features of DMH induced colonic tumours in the rat, have been described extensively. Microinvasive, polypoid and flat carcinomas occur. In rats there is little or no evidence of a polyp-to-cancer sequence as with human colonic cancer (Sunter *et al.*, 1978; Nauss *et al.*, 1984).

The histologist (RC) validated the macroscopic visual assessment of the lesions by cutting and examining sections. Because of the large number of tumours produced, an average of four tumours per rat were examined. All medium and large tumours could be accurately diagnosed grossly and all small tumours could be distinguished from lymphoid nodules, except for rare cases of tumours developing in lymphoid nodules.

Analysis of variance and corrected Chi-squared was used for analysis. The Students *t*-test was used to compare mean values. A probability of $<0.05$ was taken as significant. Ethics committee approval was obtained for this study according to the guide-lines of Australia's National Health and Medical Research Council.

### Results

Up to the final week of the study there was no signficant difference (ANOVA $P \leqslant 0.05$) between weekly consumption of diet or weight gain within or between any of the groups. In the last week some weight loss occurred among rats receiving DMH. Soft, dark yellow faeces were characteristic of the rats receiving sulphasalazine.

Although the chronological duration of administration of the carcinogen and the therapeutic drugs was concurrent, technically the therapeutic drugs were not administered simultaneously with the DMH. They were given by different routes (by subcutaneous injection for the carcinogen and orally for the drugs) and the weekly injection of DMH would have been metabolised within 24 h, whereas the therapeutic drugs were administered daily, throughout the experiment. Therefore, any competitive metabolic effect would not be expected to extend beyond the 24 h period following the weekly carcinogen injection.

Colonic adenocarcinomas occurred in all of the rats receiving DMH injections, but not in any of the rats not receiving the carcinogen. The tumours were all adenocarcinomas of varying histological type and differentiation, according to WHO classifications. The small tumours were mostly polypoid, well differentiated adenocarcinomas. A minority were intramucosal and could be defined as 'adenomas', if there was no invasion through the muscularis mucosae.

Most tumours showed at least minimal invasion into the submucosa. The architectural and cytological features were similar in those that were intramucosal and those that were invasive. Unlike human colonic carcinoma, there was no clear adenoma-cancer sequence. The 'adenomas' appeared to be small early cancers differing only by absence of invasion through the muscularis mucosae (Figures 1 and 2).

Larger diameter tumours tended to have deep invasion of muscularis propria and serosa (Figures 3 and 4). The large carcinomas also tended to contain areas of mucinous adenocarcinoma and/or poorly differentiated adenocarcinoma, including signet ring cell forms. A minority of carcinomas appeared to arise in lymphoid nodules with intact overlying normal mucosa.

Differences, in both numbers and size of tumours between groups occurred. There was a significant increase in tumour numbers (ANOVA $P \leqslant 0.05$) in the groups receiving metronidazole, sulphasalazine and 30 mg $Kg^{-1}$ $dy^{-1}$ 5-aminosalicylic acid, compared with the rats receiving DMH only (Table I). In all groups, the majority of tumours occurred in

**Figure 1** Small polypoid adenocarcinoma with micro-invasion of muscularis mucosae by single gland. Magnification × 54.

**Figure 2** Small polypoid adenocarcinoma with early invasion into submucosa. Magnification × 54.

**Figure 3** Large adenocarcinoma with invasion through muscularis propria into serosa. Magnification × 30.

**Figure 4** Usual well differentiated adenocarcinoma. Magnification × 120.

the left colon. However, the percentage of tumours, occurring in the right colon, increased significantly with sulphasalazine (Table I).

We tabulated the size of the tumours in the different groups (Table II). No tumours with a diameter greater than 10 mm were found in the group receiving DMH alone. All other groups, with the noteworthy exception of the group receiving 60 mg $Kg^{-1}$ $dy^{-1}$ 5-ASA had tumours greater than 10 mm in diameter. In addition, there was a significant proportion, of smaller tumours (< 5 mm), in the group receiving 60 mg $Kg^{-1}$ $dy^{-1}$ 5-ASA.

### Discussion

In this study, metronidazole 18 mg $Kg^{-1}$ $dy^{-1}$; sulphasalizine 60 mg $Kg^{-1}$ $dy^{-1}$ and 5-aminosalycylic acid 30 mg $Kg^{-1}$ $dy^{-1}$ exhibited a co-carcinogenic effect on the number of colonic carcinomas produced in rats receiving DMH. The results for olsalazine were equivocal and 60 mg $Kg^{-1}$ $dy^{-1}$ 5-ASA had no co-carcinogenic effect (Table I).

The 18 mg $Kg^{-1}$ $dy^{-1}$dosage of metronidazole used in these experiments is considerably less than the 50 mg-$Kg^{-1}$ $dy^{-1}$ previously reported. The lower dose is closer to the therapeutic dosage used in maintenance therapy of inflammatory bowel disease in humans. Despite this dosage reduction, there is still a significant co-carcinogenic effect with metronidazole. Further dose response studies in this model may determine if there is a minimum co-carcinogenic dosage with metronidazole.

The inclusion of metronidazole in this study and the reproducible findings, of a co-carcinogenic effect, provides a valid comparison for the results which we obtained with other drugs, not previously reported in this model. The possible mechanism of the co-carcinogenic action of metronidazole remains obscure. An effect of absorbed metabolites of metronidazole on colonic bacteria has been postulated (Speck *et al.*, 1976).

**Figure 5** Example of colonic mucosa (normal) from a rat receiving metronidazole, but no dimethylhydrazine. Magnification × 120.

**Figure 6** Example of colonic mucosa (normal) from a rat receiving olsalazine, but no dimethylhydrazine. Magnification × 120.

**Table I** Comparison of site and numbers (%) of total colonic tumours in groups receiving either dimethylhydrazine only or a therapeutic drug as well as DMH

| | | Number of tumours per group | | | | | | | | |
|---|---|---|---|---|---|---|---|---|---|---|
| | | Left colon | | Right colon | | Caecum | | Total | Mean ± s.e. | Rats in group |
| | | n | % | n | % | n | % | | n | n |
| | *mg Kg⁻¹ wk⁻¹* | | | | | | | | | |
| DMH | 40 | 49 | (70.0) | 21 | (30.0) | 0 | (0.0) | 70 | 6.4 ± 0.69 | 11 |
| | *mg Kg⁻¹ wk⁻¹* | | | | | | | | | |
| + metronidazole | 18 | 190 | (77.2)[b] | 50 | (20.3)[b] | 6 | (2.5) | 246 | 13.7 ± 1.52[a] | 18 |
| + sulphasalazine | 60 | 87 | (61.7)[b] | 54 | (38.3)[b] | 0 | (0.0) | 141 | 11.8 ± 2.16[a] | 12 |
| + olsalazine | 60 | 46 | (76.7) | 14 | (23.3) | 0 | (0.0) | 60 | 10.0 ± 2.75 | 6 |
| + 5-ASA | 30 | 73 | (75.3) | 24 | (24.7) | 0 | (0.0) | 97 | 13.9 ± 3.06[a] | 7 |
| + 5-ASA | 60 | 40 | (78.4) | 11 | (21.6) | 0 | (0.0) | 51 | 8.5 ± 3.68 | 6 |

[a]Compared to DMH alone, the mean number of tumours per rat differs significantly (*t*-test, $P < 0.05$). [b]Between metronidazole and sulphasalazine groups the proportion of tumours in the right and left colon is significant (corrected Chi-squared, df = 1, $P < 0.001$).

**Table II** Comparative size in millimetres (%) of total colonic tumours occurring in groups of rats receiving either dimethylhydrazine only or a therapeutic drug as well as DMH

| | | Number and size of tumours per group | | | | | | | | |
|---|---|---|---|---|---|---|---|---|---|---|
| | | <5 mm | | 5–9 mm | | ≥ 10 mm | | total ≥ 5 mm | | Rats in group |
| | | n | % | n | % | n | % | n | % | n |
| | mg Kg⁻¹ wk⁻¹ | | | | | | | | | |
| DMH | 40 | 48 | (68.6) | 22 | (31.4) | 0 | (0.0) | 22 | (31.4) | 11 |
| | mg Kg⁻¹ dy⁻¹ | | | | | | | | | |
| + metronidazole | 18 | 173 | (70.3) | 52 | (21.1) | 21 | (8.6)[a] | 73 | (29.7) | 18 |
| + sulphasalazine | 60 | 105 | (74.5) | 30 | (21.3) | 6 | (4.2) | 36 | (25.5) | 12 |
| + olsalazine | 60 | 39 | (65.0) | 19 | (31.7) | 2 | (3.3) | 21 | (35.0) | 6 |
| + 5-ASA | 30 | 61 | (62.9) | 22 | (22.7) | 14 | (14.4)[a] | 36 | (37.1) | 7 |
| + 5-ASA | 60 | 48 | (94.1)[a] | 3 | (5.9)[a] | 0 | (0.0) | 3 | (5.9)[a] | 6 |

[a]Compared with DMH alone the proportion of smaller (or larger) tumours is significant (corrected Chi-squared, df = 1, $P < 0.01$).

The reported distribution of colonic tumours differs according to variable study factors, such as the frequency and dosage of DMH and the rat strain used in experiments (Nauss et al., 1984; McGarrity et al., 1988). Even so, a predominance of tumours occurring in the left colon is the usual finding, therefore the shift to a right colon tumour distribution among rats receiving both DMH and sulphasalzine, in our study, may be a significant finding, which warrants further study.

Pharmokenetic studies of sulphasalazine have shown that 70%, of this drug, reaches the colon unaltered. When absorbed sulphasalazine, returned to the small intestine in bile is included, 90% of the original dosage eventually reaches the colon. In humans, from 1% to 7% of unchanged sulphasalazine may be recovered in faeces (Schroeder & Campbell, 1972).

Sulphazalazine inhibits the intestinal absorption of folate. It has been postulated that sulphasalazine may cause mucosal dysplasia secondary to folic acid deficiency (Selhub et al., 1978; Clinical Nutrition Cases, 1988). The incidence of dysplasia in patients receiving sulphasalazine, for inflammatory bowel disease, has been found to be greater than in patients not receiving the drug (Lashner et al., 1989). Sulphasalazine has been shown to be a competitive inhibitor of folate dependent enzymes. However, in our studies, rat serum folate levels were depleted due to sulphasalazine (subsequent analysis), but secondary dyplasia was not observed. These findings require further investigation.

In this study, low dosage 30 mg $Kg^{-1} dy^{-1}$ 5-ASA, appeared to be co-carcinogenic, while high dosage 60 mg-$Kg^{-1} dy^{-1}$ 5-ASA, had no such effect; indeed the higher dose inhibited tumour size. This inhibitory effect parallels the *in vitro* finding, that concentrations of 15 mM 5-ASA inhibit cancer cell growth, in human cancer cell lines (Desai et al., 1989).

Because 5-ASA is absorbed in the small bowel, only 2% of the total dose may be recovered in faeces (Nielsen & Bondesen, 1983). Nevertheless, in our experiment, doubling the dosage from 30 to 60 mg $Kg^{-1} dy^{-1}$ had a beneficial effect in the colon; as reflected in the reduction in size of colonic tumours among rats in this treatment group. Therefore, it seems appropriate to conclude that with the 60 mg $Kg^{-1} dy^{-1}$ 5-ASA regime, in rats, sufficient 5-ASA reaches the colon, to act locally on colonic mucosa.

As olsalazine is not absorbed in the small intestine, 95% reaches the colon to be split by bacteria. The concentration of 5-ASA in faeces, after administration of olsalazine, is twice that after sulphasalazine (Lauritsen et al., 1984). Considering these characteristics, of olsalazine, we are unable to explain why olsalazine was not equally as effective as 60 mg-$Kg^{-1} dy^{-1}$ 5-ASA, in inhibiting tumour size, in this animal model.

Although these results were obtained in the 1,2-dimethylhydrazine animal model, there may be a stronger association between this animal model, hydrazines and human colonic cancer, than is at first apparent. Hydrazines are potent carcinogens. Humans may be exposed to hydrazines in their environment. This chemical occurs as industrial and food contaminants (Toth et al., 1975) and is also found in tobacco (Liu et al., 1974).

In these studies, in an animal model, we have attempted to assess the role of therapeutic drugs, used in maintenance therapy of human inflammatory bowel disease, in the development of colonic cancer. The next step may seem to be to superimpose this cancer model on an animal model of inflammatory bowel disease; to assess the combined effect. However, because an inflammatory bowel disease model would require the uses of cytotoxic chemicals, the combined effects might confound, rather than simplify investigations.

From these experiments, it appears that therapeutic drugs may, under certain conditions, act as co-carcinogens. Because of the increasing tendency to use the drugs studied in long-term maintenance therapy of inflammatory bowel disease, these findings could well have clinical significance.

### References

A-KAREEM, A.M., FLEISZER, D.M., RICHARDS, G.K., SENTERMAN, M.K. & BROWN, R.A. (1984). Effect of long-term metronidazole (MTZ) therapy on experimental colon cancer in rats. *J. Surg. Res.*, **36**, 547.

CLINICAL NUTRITION CASES (1988). Sulphasalazine inhibits folate absorption. *Nutrition Rev.*, **46**, 320.

DESAI, T.K., BULL, A.W., YANAMADALA, U., MOHIER, J.A. & LUK, G.D. (1989). 5-aminosalicylic acid (5-ASA) inhibits human colon cancer cell growth and suppresses ornithine decarboxylase activity, possible through a post transcriptional mechanism. *Gastroenterology*, **96**, A120.

FISHER, E.R., PAULSON, J.D. & MORGAN, M.M. (1981). Genesis of 1,2-dimethylhydrazine induced colon cancer. A light and electron-microscopic study. *Arch. Pathol. Lab. Med.*, **105**, 29–37.

GREENSTEIN, A.J., GENNUSO, R., SACHAR, D.B. & 4 others. (1985). Extraintestinal cancers in inflammatory bowel disease. *Cancer*, **56**, 2914.

ISABELL, G. & LEVIN, B. (1988). Ulcerative colitis and colon cancer. In Luk, G.D. (ed.), *Gastroenterology Clinics of North America*, **17**, 773. Saunders: Philadelphia.

KRAUSE, J.R., AYUYANG, H.Q. & ELLIS, L.D. (1985). Occurrence of three cases of carcinoma in individuals with Crohn's disease treated with metronidazole. *Am. J. Gastro.*, **80**, 978.

LASHNER, B.A., HEIDENREICH, P.A., SU, G.L., KANE, S.V. & HANAVER, S.B. (1989). Effect of folate supplementation on the incidence of dysplasia and cancer in chronic ulcerative colitis. *Gastroenterology*, **97**, 255.

LAURITSEN, K., HANSEN, J., RYDE, M. & RASK-MADSEN, J. (1984). Colonic azodisalicylate metabolism determined by *in vivo* dialysis in healthy volunteers and patients with ulcerative colitis. *Gastroenterology*, **86**, 1496–1500.

LIU, Y., SCHMELTZ, I. & HOFFMAN, D. (1974). Chemical studies on tobacco smoke. Quantitative analysis of hydrazine in tobacco and cigarette smoke. *Anal. Chem.*, **46**, 885.

MCGARRITY, T.J., PEIFFER, L.P. & COLONY, P.C. (1988). Cellular proliferation in proximal and distal rat colon during 1,2-dimethylhydrazine induced carcinogenesis. *Gastroenterology*, **95**, 343–348.

MEYERS, S., SACHAR, D.B., PRESENT, D.H. & JANOWITZ, H.D. (1987). Olsalazine sodium in the treatment of ulcerative colitis among patients intolerant of sulfasalazine. A prospective randomized placebo-controlled, double-blind, dose-ranging clinical trial. *Gastroenterology*, **93**, 1255.

NAUSS, K.M., LOCNISKAR, M., PAVLINA, T. & NEWBERNE, P.M. (1984). Morphology and distribution of 1,2-dimethylhydrazine dihydrochloride-induced colon tumours and their relationship to gut-associated lymphoid tissue in the rat. *JNCI*, **73**, 915–924.

NIELSEN, O.H. & BONDESEN, S. (1983). Kinetics of 5-aminosalicylic acid after jejunal instillation in man. *Br. J. Clin. Pharmacol.*, **16**, 738.

ROSEN, A., URSING, B., ALM, T. & 10 others (1982). A comparative study of metronidazole and sulphasalazine for active Crohn's disease: The cooperative Crohn's Disease Study in Sweden. 1. Design and methodological considerations. *Gastroenterology*, **83**, 541.

SCHRODER, H. & CAMPBELL, D.E.S. (1972). Absorption, metabolism and excretion of salicylazosulfapyridine in man. *Clin. Pharmacol. Ther.*, **13**, 539.

SELHUB, J., DHAR, G.J. & ROSENBERG, I.H. (1978). Inhibition of folate enzymes by sulphasalazine. *J. Clin. Inv.*, **61**, 221,

SLOAN, D.A.F., LEISZER, D.M., RICHARDS, G.K., MURRAY, D. & BROWN, R.A. (1983). Increased incidence of experimental colon cancer associated with long-term metronidazole therapy. *Am. J. Surg.*, **145**, 66.

SPECK, W.T., STEIN, A.B. & ROSENKRANZ, H.S. (1976). Mutagenicity of metronidazole: presence of several active metabolites in human urine. *J. Nat. Cancer Inst.*, **56**, 283.

SUNTER, J.P., WRIGHT, N.A. & APPLETON, D.R. (1978). Cell population kinetics in the epithelium of the colon of the male rat. *Virchows Arch [B]*, **26**, 275–278.

TOTH, B. (1975). Synthetic and naturally occurring hydrazines as possible cancer causative agents. *Cancer Res.*, **35**, 3693.